# Generalized Molluscum contagiosum in an HIV infected patient

**DOI:** 10.1186/1471-2334-14-S4-P49

**Published:** 2014-05-29

**Authors:** Ovidiu Roşca, Andreea Ardeleanu, Caius Solovan

**Affiliations:** 1 Department of Infectious Diseases I, Dr. Victor Babeş University of Medicine and Pharmacy, Timişoara, Romania; 2 Department of Infectious Diseases I, Dr. Victor Babeş Clinical Hospital of Infectious Diseases and Pneumology, Timişoara, Romania; 3 Department of Dermatology, Dr. Victor Babeş University of Medicine and Pharmacy, Timişoara, Romania

## 

Molluscum contagiosum is a benign contagious disease caused by a poxvirus. In an immunocompetent host molluscum contagiosum is most frequently a self-limiting benign viral disease of the skin and rarely of the mucous membranes. Atypical forms of molluscum contagiosum may be challenging to diagnose and are found in immunocompromised patients where they indicate severe impairment of cellular immunity. We report the case of a 45-years old patient admitted in our department in January 2014, for skin-colored and violaceus, painless papules and nodules on the arms, forearms, chest, face, inguinal and genital regions; the lesions appeared about 6 months ago, on the upper limbs and progressively extended. The patient was diagnosed with HIV infection in 2011, but did not follow antiretroviral therapy and never submitted to control until January 2014. In January 2014: CD4: 40 cells/μL, viral load: 112,617 copies/mL. He received antiviral therapy with acyclovir topical local therapy and antiretroviral treatment and evolution was favorable.

